# SEVERITY OF ANXIETY AND SYMPTOMS OF DYSFUNCTIONAL BREATHING IN PATIENTS WITH CHRONIC RESPIRATORY DISEASES IN THE COVID-19 PANDEMIC

**DOI:** 10.1192/j.eurpsy.2023.1664

**Published:** 2023-07-19

**Authors:** E. R. Semenova, E. Pervichko, D. Shulga, I. Shishkova

**Affiliations:** 1 Moscow State University named after ‘M. V. Lomonosov’, Moscow; 2 Ryazan State Medical University, Ryazan, Russian Federation

## Abstract

**Introduction:**

The conditions of the COVID-19 pandemic, as well as the risks associated with it, led to a deterioration in the emotional state of many people: during the pandemic, an increase in the frequency of anxiety disorders was noted, especially among patients with chronic diseases of the respiratory system. Since the beginning of the COVID-19 pandemic, the respiratory system has been described as very “vulnerable” to the coronavirus.

**Objectives:**

To study the severity of anxiety and symptoms of dysfunctional breathing (DB) in patients with chronic respiratory diseases in the conditions of the COVID-19 pandemic.

**Methods:**

We used: «The Perceived Stress Scale-10», «State-Trait Anxiety Inventory», «Short Health Anxiety Inventory», and Nijmegen questionnaire.The sample: 89 patients with respiratory diseases, the average age was 49.21±18.3. Of these, 38 patients with upper respiratory tract diseases (URTD) (J30-J39) and 51 patients with lower respiratory tract diseases (LRTD) (J40-J47). 49 people had a history of COVID-19 disease.

**Results:**

The sample of patients with both URTD and LRTD diseases is characterized by a high level of stress (27.13±6.937 vs 28.59±6.014, p=0.293). The average indicators of health anxiety in patients with LRTD diseases significantly exceed those of patients with URTD diseases (20.18±8.881 vs 15.68±7.022; p=0.01), which is consistent with data on a greater assessment of the disease threat compared with patients with upper respiratory tract diseases.The severity of DB symptoms in the group of patients with LRTD diseases significantly exceeds that in the group of patients with URTD (19.05±12.340 vs 24.29±11.470; p=0.042). Also in this group, there is a greater severity of individual symptoms of DB: spasm of the mouth muscles (p= 0.015), accelerated deep breathing (p= 0.002), shallow breathing (p=0.001) and the inability to take a deep breath (p=0.001).When analyzing the symptoms among patients who had and did not have a history of COVID-19, significant differences were found in such manifestations of DB as dizziness (1.98±1.351 vs 1.27±1.232; p=0.024) and confusion in the environment (0.61±0.891 vs 0.18±0.465; p=0.010), and also according to the severity of the symptoms of DB in general (23.76±11.996 vs 17.81±8.461; t=2.007, p=0.043).During the analysis, a relationship was established between the severity of DB symptoms and alertness to bodily sensations (R=0.259, p=0.014), which may indicate both an increase in DB symptoms with increased attention toward sensory sensations, and greater screening activity with an increase in DB symptoms. Increased alertness to bodily sensations is associated with situational stress (R=0.530; p=0.001) and personal anxiety (R=0.495, p=0.001).

**Conclusions:**

The results obtained make it possible to identify categories of patients with chronic respiratory diseases who need psychological counseling and psychotherapy.

**Disclosure of Interest:**

None Declared

